# How does the distribution of work tasks among home care personnel relate to workload and health-related quality of life?

**DOI:** 10.1007/s00420-023-01997-2

**Published:** 2023-07-12

**Authors:** Fredrik Norström, Magnus Zingmark, Anita Pettersson-Strömbäck, Klas-Göran Sahlén, Malin Öhrling, Karin Bölenius

**Affiliations:** 1 Department of Epidemiology and Global Health, Umeå University, Umeå, Sweden; 2 Municipality of Östersund, Health and Social Care Administration, Östersund, Sweden; 3 Department of Health Sciences, Lund University, Lund, Sweden; 4 Department of Psychology, Umeå University, Umeå, Sweden; 5 Department of Nursing, Umeå University, Umeå, Sweden

**Keywords:** Sweden, Work environment, Occupational health, Health care, QPSNordic, EQ-5D

## Abstract

**Background:**

The work for Swedish home care workers is challenging with a variety of support and healthcare tasks for home care recipients. The aim of our study is to investigate how these tasks relate to workload and health-related quality of life among home care workers in Sweden. We also explore staff preferences concerning work distribution.

**Methods:**

A cross-sectional study was conducted in 16 municipalities in Northern Sweden. Questionnaires with validated instruments to measure workload (QPSNordic) and health-related quality of life (EQ-5D), were responded by 1154 (~ 58%) of approximately 2000 invited home care workers. EQ-5D responses were translated to a Quality-adjusted life-year (QALY) score. For 15 different work task areas, personnel provided their present and preferred allocation. Absolute risk differences were calculated with propensity score weighting.

**Results:**

Statistically significantly more or fewer problems differences were observed for: higher workloads were higher among those whose daily work included responding to personal alarms (8.4%), running errands outside the home (14%), rehabilitation (13%) and help with bathing (11%). Apart from rehabilitation, there were statistically significantly more (8–10%) problems with anxiety/depression for these tasks. QALY scores were lower among those whose daily work included food distribution (0.034) and higher for daily meal preparation (0.031), both explained by pain/discomfort dimension. Personnel preferred to, amongst other, spend less time responding to personal alarms, and more time providing social support.

**Conclusion:**

The redistribution of work tasks is likely to reduce workload and improve the health of personnel. Our study provides an understanding of how such redistribution could be undertaken.

**Supplementary Information:**

The online version contains supplementary material available at 10.1007/s00420-023-01997-2.

## Background

In﻿ Sweden, home care services are focused on the personal care of home care recipients and performing household chores (Meagher et al. 2016). Those who work in home care services perform a variety of tasks both in the home (e.g., cleaning) and outside (e.g., shopping), they assist with the activities of daily life and provide care in the home in the form of delegated tasks related to drugs and rehabilitation measures. Recent decades have seen major restructuring of Swedish home care services, as demonstrated in surveys conducted in 2005 and 2015 (Strandell 2020). During the period between these surveys, administrative tasks and support for personal hygiene increased, while less time was devoted to cleaning the home care recipients’ home. While both surveys revealed great difficulties in completing all assigned tasks, this had increased from 36 per cent of personnel reporting difficulties in 2005 to 40 per cent in 2015.

The average number of home care recipients that a Swedish home care worker meets each day has increased from 4.0 in the 1980s (Szebehely et al. 2017) to 8.6 in 2005 to 11.8 in 2015 (Strandell 2020). Although structural workforce changes in Swedish home care are documented, to the best of our knowledge, no previous study has investigated the distribution of work tasks among home care workers and how this impacts their workload and health.

Home care services in Sweden are mainly provided by municipalities and financed by taxes. Individual citizens apply for home care services and a formal needs assessment is conducted by a care manager pursuant to the Swedish Social Services Act (Parliament Sweden 2022b). The municipality is then required to provide any necessary services. There is no uniformity in the services provided or in how they are provided; for example, cleaning or other household chores may be outsourced.

Swedish home care services are provided by assistant nurses, nursing assistants and staff with no formal training. While assistant nurses are expected to have completed the health and social care line in upper secondary school, or the equivalent in adult education, there is no such requirement for nursing assistants (Parliament Sweden 2019). Although there are no official statistics, it is estimated that in 2020, there were 128,985 assistant nurses working within Sweden’s home care sector and 77,003 nursing assistants working across all sectors (Statistics Sweden 2018). Of these nursing assistants, 34,145 were employed by a municipality, the local authority responsible for home care in Sweden (Swedish Association of Local Authorities and Regions 2022). Previously, a quantitative and a qualitative study in Sweden have highlighted some of the challenges associated with lack of education (Hasson and Arnetz 2008; Swedberg et al. 2013), yet little is known about the relationship between level of education of home care workers and the allocation of work tasks and how this impacts personnel.

In Sweden, political reforms since the 1990s have resulted in a choice-based health and welfare system with both public- and private-sector providers. Based on the principles of New Public Management, these reforms were intended to move the labour market in a more service-oriented direction (Smith and Rauhut 2019), presumably leading to more cost-effective health and social care (Andersson and Kvist 2015). As a consequence of the community care reform of 1992 (commonly known as *Ädelreformen*) (Szebehely and Trydegård 2012), there has been a notable shift from hospital-based healthcare towards care provided in patients’ own homes. Many reports have highlighted the impact of restructuring in Swedish working life resulting in an increased workload for many occupations (Falkstedt and Hemmingsson 2011; Forsberg Kankkunen et al. 2014; Szebehely et al. 2017). It is estimated that anything from a third to half of all personnel working in home care services in the Nordic countries are considering resigning due to the challenging working conditions, with the highest rates reported in Sweden (Trydegård 2012; Van Aerschot et al. 2021).

Sweden is not the only country undergoing structural changes in home care (Strandell 2020), similar trends are also visible in Norway (Grønoset Grasmo et al. 2021a) and Switzerland (Möckli et al. 2020), countries from which reports of challenging workloads have also emerged (Andersen and Westgaard 2015; Möckli et al. 2020). One qualitative study of Norwegian home care workers suggests that unpredictable working conditions have a negative impact on safety, health and wellbeing (Grønoset Grasmo et al. 2021a), underlining the need to adapt the distribution of work to the expectations and needs of personnel. As previously reported in our own study, high workloads are associated with a reduction in health-related quality of life among home care workers (Sjöberg et al. 2020). In addition, high workloads among home care workers have been shown to lead to chronic fatigue syndrome and other negative health consequences (Hasson and Arnetz 2008; Skovdahl et al. 2008), as well as higher rates of sick leave (Försäkringskassan 2018; Horneij et al. 2004). Studies in Sweden (Sjöberg et al. 2020) and Switzerland (Möckli et al. 2020) have indicated that strong social support protects good health. Previous research has not thoroughly investigated how the allocation of work tasks could affect the work environment of home care workers. Such information is of importance not only for a better understanding of potential consequences of reducing resources for home care workers, but also for getting a better understanding on how to handle the expected future increased demand of home care. There is also a lack of information on the mix of work tasks that home care workers prefer, and potentially could some of the work tasks be reallocated to other expertises.

The aim of our study is to understand how different work tasks are related to workload and health-related quality of life among home care workers in Sweden. Furthermore, our study aims to contribute a better understanding of the preferred distribution of work tasks according to home care workers themselves. Home care services varies much between countries, but as previous literature has shown similarities between Sweden and other countries, our study is expected to still provide valuable insights to other countries.

## Methods

### Design

A cross-sectional study was undertaken using the 12-page questionnaire *A study of the work environment in home care services*. This included parts of the General Nordic Questionnaire for Psychological and Social Factors at Work (QPSNordic), which is a validated instrument covering psychosocial and social factors at work (Dallner et al. 2000), and the EuroQol 5 dimensions (EQ-5D), which is a validated instrument to measure health-related quality of life (Devlin and Brooks 2017). The questionnaire included 15 work tasks covering social services, social support, cleaning, meal preparation, and delegated tasks related to drugs. Current study is part of the same project as a previous publication (Sjöberg et al. 2020). The same survey as in that study that focused on the relationship between workload and health-related quality of life is used in current study.

### Participants and data collection

In our study, we investigate the situation for home care workers in three northern Swedish counties, including assistant nurses, nursing assistants and personnel with no formal healthcare training. All 30 municipalities in these counties were invited to participate and 16 agreed to do so.

In October 2017, the study team delivered the 12-page questionnaire to participating municipalities for distribution to their employees. We instructed a contact person to distribute the questionnaire to personnel who are members of a working group and, therefore, have sufficient work experience. In most cases staff responded to questionnaires during workplace meetings. A total of around 2,000 home care workers (approximate number provided by the municipalities) were invited to respond to the questionnaire, of whom 1,154 responded before the end of January 2018, a response rate of 58%.

Study participants were informed about the purpose of the study and the handling of personal data, including confidentiality, on page 2 of the questionnaire. Participants consented to participate when they submitted their questionnaire. The Regional Ethical Review Board in Umeå, Sweden, approved the survey (Dnr 2016–68-31 M).

### Variables

QPSNordic is a validated instrument that has been used both in workplace settings and research since 2000 (Dallner et al. 2000; Nordic Council of Ministers). The full version of QPSNordic consists of 129 questions and from these question also a shorter version with 37 questions is presented (Dallner et al. 2000). In our questionnaire, we included the 27 questions in the shorter version that were related to psychosocial and social factors at work. The four questions belonging to the two subscales *Quantitative-, and Learning demands* and *Job demands* were used to construct a workload variable in current study. The questions were: ‘Is your workload irregular so that the work piles up?’; ‘Do you have too much (work) to do?’; ‘Are your work tasks too difficult for you?’ and ‘Does your job require that you acquire new knowledge and new skills’. There were five response options: ‘very seldom or never’, ‘rather seldom’, ‘sometimes’, ‘rather often’ and ‘very often or always’. We defined individuals as having a high workload if the median of the four workload questions were at least ‘sometimes’ and treated it as exposed in analysis; otherwise, individuals were defined as having a normal workload. In a recent study from the same dataset (Sjöberg et al. 2020), we defined the cut-off of workload also based on a mean value of 2.5 respectively 3.0, but considered current definition to be the most appropriate. In the present study, we are only interested in aggregated workload, not the individual responses themselves.

EQ-5D is a validated, generic non-disease-specific instrument used to measure health-related quality of life (Devlin and Brooks 2017). The EQ-5D 3L version consists of a descriptive system with five dimensions of health and a visual analogue scale (EQ-VAS), which ranges from 0 to 100. The five dimensions are mobility, self-care, usual activities, pain/discomfort, and anxiety/depression. Each dimension includes questions with three response alternatives corresponding to no, some, and extreme problems. The responses to the descriptive system were translated into a quality-adjusted life year (QALY) score with the anchor points 0 (death) and 1 (full health), using the United Kingdom value set (Dolan 1997).

For analyses of the health dimensions of EQ-5D themselves, responses were dichotomised to ‘no problems’, and ‘problems’, with the latter including responses corresponding to some and extreme problems. We restricted these analyses to the pain/discomfort and anxiety/depression dimensions as only 5 to 63 respondents reported some or extreme problems with the other dimensions (see Table 3 in Sjöberg et al. (Sjöberg et al. 2020) for complete responses to the EQ-5D dimensions). With such few responses and a sub-analysis required for each work task, we considered the risk of misleading conclusions to be too high.

Two questions were related to the various work tasks performed by home care workers. Informed by a previous study in a participating municipality (Zingmark and Norström 2021), 15 work tasks were derived from this municipality’s 43 different decisions regarding support allocated by a care manager based on the Social Services Act (Parliament Sweden 2022b) (Table 2). In the first question, referred to here as *current work distribution*, the question was: ‘Estimate how often you perform the following actions in your daily work’ For the current work distribution question, the response alternatives ‘multiple times per day’ and ‘one to two times per day’ were defined as ‘daily’, while all other responses (i.e., ‘at least once a week’, ‘at least once a month’ and ‘not at all/in case of urgent need’) were defined as ‘not daily’. The second question, referred to as *future work distribution*, was: ‘To what extent would you like to perform the following tasks?’. The response alternatives were ‘more’, ‘equal’, ‘less’ and ‘not at all’.

The variables gender, healthcare training, marital status, and tenure in home care were used as covariates in our analyses, as we considered them to potentially confound some of our analyses. ‘Woman’ was used as exposure for gender. Respondents were asked about their healthcare training, the outcomes ‘assistant nurse’ and ‘other healthcare training, specify’ were defined as ‘assistant nurse’ and used as a reference group, while ‘nursing assistant’ and ‘no formal health education’ were defined as ‘other education’ and used as exposure. In our presentation of current and future work distribution, ‘assistant nurse’ and ‘no formal health education’ were presented separately. The marital status categories were either ‘single’, used as exposure, or ‘cohabiting’, where ‘single’ consisted of all those who were single or single parents. The categories for tenure in home care were ‘less than one year’, ‘one to five years’, ‘six to fifteen years’ and ‘more than fifteen years’. Based on age and tenure, a composite variable was derived whereby at least 5 years working in home care (regardless of age) was the exposure. Those who had worked in home care for over 5 years were divided into three groups: ‘35 years of age or under’; ‘36–54 years of age’ and ‘55 years of age or over’. The age groups were divided into three age groups where the median was within the middle group.

### Statistics

Descriptive statistics were used to present the characteristics of the sample. Pearson’s $\chi^{2}$ -test and Student’s t-test were used to test association between variables. Interactions between variables were not considered in any of our analyses. There was no observed collinearity between variables and so all candidate variables were retained for the analyses.

For the analyses in which daily work tasks was the exposure and health-related quality of life or workload the outcome, home care workers had to have worked at least 30 h a week. This inclusion criterion was used, because a lower number of working hours reduces the likelihood of performing work tasks frequently and because of the risk that results for workload and health would mainly be attributable to differences in the number of hours worked by respondents. Furthermore, responses to all covariates listed in the previous sub-section were required in multivariable analyses. Analyses of the relationship between work tasks and outcome variables, therefore, included: EQ-5D descriptive system (inclusive QALY) 788–809 respondents, EQ-VAS 761–780 respondents and workload 785–806 respondents. Missing or non-valid responses were: 44 for any of the EQ-5D questions, 44 for any of the questions used to define workload, 7 for gender, 24 for education, 11 for marital status, 6 for tenure and 19 for birth year (used to derive age). Requiring responses for covariates reduced the sample size by at most 40 participants (only 4.2% of participants with valid responses to EQ-5D and workload questions) in multivariable analyses.

Propensity score weighting was used to estimate effects on the outcome from the main exposure. The abovementioned covariates were included in logistic regression to derive propensity scores (Lunceford and Davidian 2004). Using propensity scores in our study, results in a quasi-experimental approach. Results are also given for logistic regression showing crude and adjusted odds ratios with 95% confidence intervals. Our inverse probability weighting (IPW) estimator was derived as suggested by Lunceford and Davidian (Lunceford and Davidian 2004), and the formula for it is available in our previous publication from the project (Sjöberg et al. 2020). This estimator measures the risk difference through the use of counterfactual arguments, i.e. the effect on health if individuals are exposed. The standardised difference was calculated, both with and without weighting, to assess the balance of covariates between exposure and reference group for each covariate (Austin and Stuart 2015; Norström et al. 2017). A more detailed description of our method is included in a previous publication (Norström et al. 2017).

Stata 13.1 (StataCorp, College Station, TX) was used for the descriptive analyses. R Studio was used for confirmatory analyses (R Studio, Boston, MA), with its GLM procedure used for logistic regression, where confidence intervals were derived with the profile likelihood (R Core Team 2015). The Bootstrap technique with replacement was used to derive the mean square error, confidence intervals, which corresponded to the 2.5% and 97.5% percentiles, and p-values from 1,000 replicates (Davison and Hinckley 1997). Statistical significance was defined at the 5% level and no adjustments were done to consider the many comparisons conducted in our study.

## Results

Of 1,154 responders, 875 (76%) worked at least 30 h a week, 229 (24%) worked fewer hours and 50 gave no information about their working hours (Table 1). Most respondents were women (84%) and had worked for more than 5 years in the occupation (59%). There were 765 (71%) assistant nurses, 131 (12%) home care aides and 185 (17%) with no formal health training, and the mean age of participants was 43.9 years. A higher proportion of men (84%) than women (79%) worked at least 30 h a week. Across tenure and healthcare training, there were statistically significant differences in the percentage of home care workers committing at least 30 h a week, compared to others who worked less hours.

**Table 1:** Characteristics of the study population (*n* = 1104)^a^

	Working at least 30 h a week (*n* = 875)			Working at most 30 h a week (*n* = 229)		
	*n*		%	*n*		%
Gender						
Man (*n* = 172)	144		84	28		16
Woman (*n* = 926)	727		79	199		21
Marital status						
Married (*n* = 753)	594		79	159		21
Single (*n* = 351)	275		80	67		20
Tenure*						
< 1 year (*n* = 116)	58		62	36		38
1–5 years (*n* = 323)	244		71	71		23
6–15 years (*n* = 239)	273		62	62		19
> 15 years (*n* = 403)	297		84	58		16
Healthcare training*						
Assistant nurse (*n *= 765)	639		84	126		16
Home care aides (*n* = 131)	94		72	37		28
No formal health education (*n* = 185)	123		66	62		34
Employment form*						
Permanent (*n* = 938)	784		84	154		16
Temporary (*n* = 75)	51		68	23		32
By the hour (*n* = 89)	39		44	50		56
Tenure and age*						
Up to 5 years of experience (*n* = 403)	297		74	106		26
More than 5 years of experience and ≤ 35 years of age (*n* = 116)	93		80	23		20
More than 5 years of experience and 36–54 years of age (*n* = 323)	281		87	42		13
More than 5 years of experience and ≥ 55 years of age (*n* = 239)	187		78	52		22
Age	Mean	Median	SD^b^	Mean	Median	SD^b^
	43.9	46	13.1	42.5	44	15.5

^a^50 respondents did not provide information about hours worked during a week and was therefore excluded from this table

^b^Standard deviation

*Significance at 5% level using χ^2^ or t-test

### Distribution of work tasks

The most common tasks that home care workers regularly assisted home care recipients with were toilet visits, dressing, food distribution, delegated tasks related to drugs and supervision, tasks that at least 78% of respondents performed at least daily (Table 2). The daily work tasks that the home care workers undertook least frequently were running errands outside the home (22%), walking (22%) and accompaniment (11%).

**Table 2:** Distribution of work tasks among home care personnel (*n* = 875)

Work task	Multiple times per day	1–2 times per day	At least once a week	At least once a month	Not at all/in case of urgent need
Responding to personal alarm (*n* = 856)	134 (16%)	172 (20%)	304 (36%)	179 (21%)	67 (7.8%)
Running errands outside the home (errands, purchases, etc.) (*n* = 865)	52 (6%)	141 (16%)	514 (59%)	92 (11%)	66 (7.6%)
Domestic chores in the home (cleaning, washing, making the bed, etc.) (*n* = 868)	52 (6.0%)	141 (16%)	514 (59%)	92 (11%)	66 (7.6%)
Social support (*n* = 859)	270 (31%)	195 (23%)	211 (24%)	29 (3.3%)	35 (4.0%)
Help at toilet visits (*n* = 866)	472 (55%)	256 (30%)	92 (11%)	30 (3.5%)	16 (1.8%)
Dressing (*n* = 868)	496 (57%)	277 (32%)	71 (8.2%)	9 (1.0%)	15 (1.7%)
Food distribution (*n* = 862)	462 (54%)	213 (25%)	92 (11%)	15 (1.7%)	80 (9.3%)
Meal preparation (*n* = 864)	196 (23%)	218 (25%)	186 (22%)	84 (9.7%)	180 (21%)
Feeding, practical help with meals (*n* = 858)	148 (17%)	245 (29%)	200 (23%)	60 (7%)	205 (24%)
Rehabilitation measures (*n* = 857)	122 (14%)	215 (25%)	345 (40%)	91 (11%)	84 (9.8%)
Delegated tasks related to drugs (*n* = 866)	743 (86%)	69 (8.0%)	24 (2.8%)	17 (2.0%)	13 (1.5%)
Supervision (*n* = 857)^a^	500 (58%)	217 (25%)	104 (12%)	21 (2.5%)	15 (1.8%)
Accompaniment (*m* = 845)	35 (4.1%)	54 (6.4%)	200 (24%)	318 (38%)	238 (28%)
Walking (*n* = 869)	52 (6.0%)	142 (16%)	518 (60%)	107 (12%)	50 (5.8%)
Help with bathing (*n* = 867)	97 (11%)	236 (27%)	459 (53%)	35 (4.0%)	40 (4.6%)

^a^Supervision refer to a visit or contact by telephone one or more times per day to ensure that the home care recipient is well

Here were 6 to 30 participants who failed to give a valid response to the questions

### Work tasks and workload

There were statistically significant higher workloads for those undertaking these daily tasks than other respondents: responding to personal alarms (8.4% more, *p* = 0.002), running errands outside the home (14% more, *p* < 0.001), rehabilitation measures (13% more, *p* < 0.001) and help with bathing (11% more, *p* < 0.001) (Table 3). None of the work tasks were statistically associated with a lower workload. Analyses conducted with logistic regression showed the same pattern. The responses to the work task and workload questions are presented in Table S1 in Appendix 1.

**Table 3:** Relationship between workload and work task

Work task		OR-crude	OR–adjusted^a^	Risk difference^b^	*p*
Responding to personal alarm (*n* = 792)	Daily	1.48 (1.02–2.14)	1.55 (1.12–2.15)	0.084 (0.017–0.154)	0.002
	Not daily		1		
Running errands outside the home (*n* = 802)	Daily	2.54 (1.72–3.74)	1.95 (1.37–2.78)	0.143 (0.065–0.219)	< 0.001
	Not daily		1		
Domestic chores in the home (*n* = 804)	Daily	1.42 (0.95–2.17)	1.19 (0.85–1.68)	0.036 (− 0.034–0.104)	0.288
	Not daily		1		
Social support (*n* = 795)	Daily	1.26 (0.87–1.83)	1.29 (0.94–1.77)	0.050 (− 0.012–0.114)	0.128
	Not daily		1		
Help at toilet visits (*n* = 802)	Daily	1.21 (0.73–2.07)	1.08 (0.71–1.68)	0.015 (− 0.079–0.100)	0.718
	Not daily		1		
Dressing (*n* = 803)	Daily	2.67 (1.29–6.47)	2.00 (1.14–3.72)	0.111	n/a
	Not daily		1		
Food distribution (*n* = 801)	Daily	1.05 (0.68–1.65)	0.88 (0.60–1.29)	− 0.044 (− 0.122–0.039)	0.296
	Not daily		1		
Meal preparation (*n* = 800)	Daily	1.42 (0.99–2.04)	1.02 (0.75–1.40)	0.005 (− 0.059–0.065)	0.886
	Not daily		1		
Feeding, practical help with meals (*n* = 797)	Daily	1.61 (1.11–2.32)	1.20 (0.88–1.65)	0.038 (− 0.029–0.100)	0.220
	Not daily		1		
Rehabilitation measures (*n* = 795)	Daily	2.05 (1.42–2.97)	1.88 (1.37–2.59)	0.129 (0.068–0.191)	< 0.001
	Not daily		1		
Delegated tasks related to drugs (*n* = 806)	Daily	0.66 (0.35–1.36)	0.74 (0.41–1.38)	− 0.077	n/a
	Not daily		1		
Supervision (*n* = 797)	Daily	1.00 (0.61–1.68)	1.02 (0.67–1.58)	0.004	n/a
	Not daily		1		
Accompaniment (*n* = 785)	Daily	1.25 (0.69–2.15)	0.97 (0.57–1.59)	− 0.023 (− 0.125–0.084)	0.652
	Not daily		1		
Walking (*n* = 785)	Daily	1.86 (1.24–2.76)	1.35 (0.93–1.94)	0.050 (− 0.024–0.127)	0.212
	Not daily		1		
Help with bathing (*n* = 803)	Daily	2.62 (1.82–3.80)	1.73 (1.26–2.39)	0.105 (0.039–0.172)	< 0.001
	Not daily		1		

^a^A relative risk above 1 means more problems with health-related quality of life when conducting daily support with the work task

^b^A risk difference above 0 means more problems with health-related quality of life when conducting daily support with the work task

n/a Too few who conducts the task daily or not daily to get valid confidence intervals and *p*-values

### Work tasks and health-related quality of life

There were statistically significant effects on QALY for those who provided daily support than other respondents in the form of food distribution, with a 0.034 lower QALY score (*p* = 0.032), and meal preparation, with 0.031 higher QALY score (*p* = 0.016). There were no statistically significant differences in EQ-VAS associated with performing a task on a daily basis (Table 4). For the EQ-5D pain/discomfort dimension, there were statistically significantly more problems among home care workers who provided daily support in the form of food distribution (13%, *p* = 0.002) and delegated tasks related to drugs (25%, *p* < 0.001) compared with others. Meal preparation at least once a day, on the other hand, was associated with statistically significantly fewer reported problems (9.4%, *p* = 0.006). Statistically significantly more home care workers who responded to personal alarms (10%, *p* = 0.006), ran errands outside the home (10% more, *p* = 0.002) and helped with bathing (7.6%, *p* = 0.020), on a daily basis reported problems in the EQ-5D anxiety/depression dimension compared with others.

**Table 4:** Effect of health-related quality of life related to work tasks within homecare

	Quality-adjusted life years (*n* = 788–809)			EuroQol visual analogue scale (*n* = 761–780)		
	Effect^a^	Confidence interval	*p*	Effect^a^	Confidence interval	*p*
Responding to personal alarm	−0.011	− 0.036–0.014	0.368	− 0.20	− 2.91–2.28	0.936
Running errands outside the home	− 0.023	− 0.057–0.010	0.176	− 1.30	− 5.39–3.50	0.538
Domestic chores in the home	− 0.010	− 0.036–0.017	0.456	0.68	− 1.81–3.61	0.626
Social support	− 0.007	− 0.033–0.016	0.572	1.05	− 1.57–3.68	0.452
Help at toilet visits	0.006	− 0.028 – 0.043	0.738	1.97	− 0.98–4.93	0.212
Dressing	0.019	− 0.027 – 0.066	0.432	3.49	− 0.06–7.58	0.056
Food distribution	− 0.034	− 0.062 to − 0.003	0.032	− 1.46	− 3.95–1.54	0.318
Meal preparation	0.031	0.006–0.055	0.016	1.47	− 1.02–4.25	0.234
Feeding, practical help with meals	− 0.004	− 0.030–0.020	0.736	0.57	− 2.09–3.39	0.720
Rehabilitation measures	− 0.014	− 0.039–0.012	0.296	− 0.30	− 3.31–3.09	0.810
Delegated tasks related to drugs	− 0.039	− 0.091–0.019	0.188	0.87	− 4.02–5.45	0.756
Supervision	− 0.013	− 0.045–0.019	0.446	0.89	− 2.21–4.28	0.538
Accompaniment	0.002	− 0.044–0.046	0.934	0.08	− 4.24–4.20	0.950
Walking	0.002	− 0.029–0.032	0.916	0.16	− 3.46–4.30	0.968
Help with bathing	− 0.013	− 0.038–0.012	0.306	0.46	− 2.56–4.38	0.786

	Pain/Discomfort (*n* = 788–809)^b^			Anxiety/depression (*n* = 788–809)^b^		
	Effect^c^	Confidence interval	*p*	Effect^c^	Confidence interval	*p*
Responding to personal alarm	− 0.006	− 0.076–0.064	0.824	0.099	0.029–0.166	0.006
Running errands outside the home	0.004	− 0.080–0.082	0.968	0.101	0.025–0.180	0.002
Domestic chores in the home	0.037	− 0.032–0.112	0.322	0.044	− 0.022–0.111	0.208
Social support	0.016	− 0.038–0.082	0.504	− 0.022	− 0.087–0.041	0.488
Help at toilet visits	− 0.011	− 0.011–0.083	0.796	− 0.008	− 0.108–0.075	0.938
Dressing	− 0.051	− 0.181–0.081	0.410	0.044	− 0.071–0.159	0.450
Food distribution	0.131	0.047–0.214	0.002	0.022	− 0.062–0.100	0.604
Meal preparation	− 0.094	− 0.164 to − 0.021	0.006	0.011	− 0.049–0.074	0.716
Feeding, practical help with meals	0.056	− 0.012–0.127	0.118	0.006	− 0.057–0.066	0.856
Rehabilitation measures	0.067	− 0.005–0.135	0.060	0.019	− 0.042–0.087	0.514
Delegated tasks related to drugs	0.252	0.108–0.392	< 0.001	0.033	− 0.107–0.160	0.634
Supervision	0.069	− 0.025–0.171	0.134	0.047	− 0.045–0.131	0.286
Accompaniment	0.000	− 0.130–0.117	0.976	0.036	− 0.082–0.157	0.540
Walking	− 0.002	− 0.086–0.084	0.752	0.041	− 0.003–0.115	0.296
Help with bathing	0.009	− 0.062–0.074	0.772	0.076	0.011–0.143	0.020

Propensity scores were derived using gender, education level, marital status, and a variable combining age and tenure in occupation

^a^A risk difference above 0 means a poorer QALY or VAS when conducting daily support with the work task compared with others

^b^EuroQol 5 dimensions. Responses dichotomized to no problems and at least moderate problems. Problems with each of the dimensions were: 504 (59%) for pain/discomfort and 236 (27%) for anxiety/depression

^c^A risk difference below 0 means more problems with health-related quality of life when conducting daily support with the work task compared with others

### Current and future work distribution

Table 5 shows responses regarding the frequency of current and future preferred work distribution for each work task category. For each work task, results for future preferred work distribution (more, same or less) are presented for healthcare training, i.e. assistant nurse, nursing assistant and no formal education, and current work distribution (daily or not).

**Table 5:** Relationship between current distribution and future distribution (*n* = 856^a^)

Work task	Healthcare training	Work task frequency	More		Same intensity		Less		Not at all		Total	Part within occupation (%)
			*n*	%	*n*	%	*n*	%	*n*	%		
Responding to personal alarm	Assistant nurse	Daily	5	2.2	110	47	94	41	23	9.9	232	39
		Not daily	12	3.3	227	62	80	22	50	14	369	61
	Nursing assistant	Daily	1	3.6	14	50	8	29	5	18	28	33
		Not daily	3	5.1	27	47	13	22	15	26	58	67
	No formal health education	Daily	2	7.7	6	23	16	62	2	7.7	26	22
		Not daily	4	4.4	54	59	22	24	11	12	91	78
Running errands outside the home	Assistant nurse	Daily	7	4.7	86	58	40	27	16	11	149	24
		Not daily	28	6.0	292	63	68	15	79	17	467	76
	Nursing assistant	Daily	2	14	7	50	6	27	0	–	14	16
		Not daily	6	7.9	44	58	13	17	13	17	76	84
	No formal health education	Daily	1	4.1	14	58	7	29	2	8.3	24	20
		Not daily	8	8.4	66	70	18	19	3	3.2	95	80
Domestic chores in the home	Assistant nurse	Daily	11	2.6	221	51	140	33	58	13	430	70
		Not daily	7	3.8	86	47	41	22	50	27	184	30
	Nursing assistant	Daily	3	5.3	32	56	17	30	5	8.8	57	62
		Not daily	0	–	20	57	10	29	5	14	35	38
	No formal health education	Daily	5	6.2	44	55	27	34	4	5.0	80	67
		Not daily	1	2.6	22	56	13	33	3	7.7	39	33
Social support	Assistant nurse	Daily	135	41	175	53	20	6.0	3	0.9	333	54
		Not daily	118	42	144	51	16	5.6	6	2.1	284	46
	Nursing assistant	Daily	17	43	19	48	3	7.5	1	2.5	40	46
		Not daily	17	36	24	51	6	13	0	–	47	54
	No formal health education	Daily	28	40	38	54	4	5.7	0	–	70	60
		Not daily	21	45	25	53	1	2.1	0	–	47	40
Help at toilet visits	Assistant nurse	Daily	10	1.9	458	87	54	10	6	1.1	528	85
		Not daily	0	–	78	85	11	12	9	1.5	92	15
	Nursing assistant	Daily	3	4.0	61	81	8	11	3	4.0	75	82
		Not daily	0	–	13	81	2	12	1	6.2	16	18
	No formal health education	Daily	2	2.2	71	78	17	19	1	1.1	91	78
		Not daily	1	4.0	18	72	6	24	0	–	25	22
Dressing	Assistant nurse	Daily	11	2.0	495	90	41	7.4	4	0.7	551	89
		Not daily	1	1.4	54	78	7	10	7	10	69	11
	Nursing assistant	Daily	2	2.5	72	89	6	7.4	1	1.2	81	90
		Not daily	0	–	8	89	0	–	1	11	9	10
	No formal health education	Daily	2	1.9	93	87	11	10	0	–	107	90
		Not daily	1	8.3	11	92	0	–	0	–	12	10
Food distribution	Assistant nurse	Daily	11	2.3	389	83	49	10	22	4.7	471	77
		Not daily	7	4.9	84	58	17	12	36	25	144	23
	Nursing assistant	Daily	3	4.5	55	82	8	12	1	1.5	67	80
		Not daily	0	–	13	76	0	-	4	23	17	20
	No formal health education	Daily	3	2.9	86	82	12	11	4	3.8	105	88
		Not daily	4	29	9	64	0	–	1	7.1	14	12
Meal preparation	Assistant nurse	Daily	35	12	196	66	49	16	17	5.7	297	48
		Not daily	39	12	139	43	53	17	89	28	320	52
	Nursing assistant	Daily	5	11	31	67	8	17	2	4.3	46	53
		Not daily	7	17	14	34	13	31	7	17	41	47
	No formal health education	Daily	10	19	33	61	6	17	2	3.7	54	46
		Not daily	7	11	32	50	7	11	18	28	64	54
Feeding, practical help with meals	Assistant nurse	Daily	35	13	193	71	33	12	9	3.3	270	44
		Not daily	20	5.8	239	69	34	10	51	15	344	56
	Nursing assistant	Daily	6	13	30	64	7	15	4	8.5	47	52
		Not daily	1	2.3	22	50	11	25	10	23	44	48
	No formal health education	Daily	9	16	41	72	5	8.9	2	3.5	57	49
		Not daily	5	8.3	39	65	13	22	3	5.0	60	51
Rehabilitation efforts	Assistant nurse	Daily	72	29	148	60	24	9.7	4	1.6	248	40
		Not daily	98	27	215	59	33	9.0	21	5.7	367	60
	Nursing assistant	Daily	6	17	22	61	6	17	2	5.6	43	44
		Not daily	7	13	36	67	6	11	5	9.2	54	56
	No formal health education	Daily	7	17	26	63	6	15	2	4.9	41	35
		Not daily	11	15	53	71	10	13	1	1.3	75	65
Delegated tasks related to drugs	Assistant nurse	Daily	128	22	428	73	20	3.4	7	1.2	583	94
		Not daily	5	14	25	69	4	11	2	5.6	36	5.8
	Nursing assistant	Daily	11	13	67	82	2	2.4	2	2.4	82	90
		Not daily	3	33	5	86	1	11	0	–	9	10
	No formal health education	Daily	9	8.0	97	86	5	4.4	2	1.8	113	95
		Not daily	1	17	5	83	0	–	0	–	6	5.0
Supervision	Assistant nurse	Daily	68	13	413	79	36	6.9	6	1.1	523	85
		Not daily	10	11	69	78	6	6.7	4	4.5	89	15
	Nursing assistant	Daily	11	16	53	77	3	4.4	2	2.9	69	80
		Not daily	1	5.9	14	82	2	12	0	-	17	20
	No formal health education	Daily	14	15	77	80	4	4.2	1	1.0	96	81
		Not daily	7	30	15	65	1	4.3	0	–	23	19
Accompaniment	Assistant nurse	Daily	7	11	51	80	4	6.3	2	3.1	64	11
		Not daily	88	17	319	60	68	13	54	10	529	89
	Nursing assistant	Daily	3	38	5	62	0	–	0	–	8	8.8
		Not daily	17	20	48	58	11	13	7	8.4	83	91
	No formal health education	Daily	3	23	9	69	0	–	1	7.7	13	11
		Not daily	20	19	67	64	12	11	6	5.7	105	89
Walking	Assistant nurse	Daily	35	23	105	70	9	6.0	2	1.3	151	24
		Not daily	141	30	283	60	21	4.5	26	5.5	471	76
	Nursing assistant	Daily	4	29	9	64	1	7.1	0	–	14	15
		Not daily	23	29	48	61	6	7.6	2	2.5	79	85
	No formal health education	Daily	5	21	16	67	2	8.3	1	4.2	24	20
		Not daily	35	37	54	57	6	6.3	0	–	95	80
Help with bathing	Assistant nurse	Daily	17	6.7	194	76	38	15	5	2.0	254	41
		Not daily	24	6.5	290	79	35	9.5	20	5.4	369	59
	Nursing assistant	Daily	3	8.8	25	74	6	18	0	–	34	37
		Not daily	3	5.3	41	72	9	16	4	7.0	57	63
	No formal health education	Daily	4	14	21	72	3	10	1	3.4	29	20
		Not daily	6	6.9	64	74	15	17	2	2.3	116	80

^a^There were no valid answer on education for 19 participants. For work tasks, there were 22 to 78 participants who did not respond to either the question about current or future work distribution

Regardless of healthcare training and current work distribution, home care workers commonly expressed the desire for more time to provide social support, rehabilitation measures (especially among assistant nurses), walking, meal preparation, supervision and accompaniment. For social support, it varied from 36 to 45 per cent requesting more time regardless of healthcare training and current work distribution, while also for the other mentioned work tasks, at least 10 per cent in these groups requested more time. Regardless of healthcare training and current work distribution, over 20 per cent of home care workers expressed the desire to spend less time responding to personal alarms, performing domestic chores in the home and running errands outside the home. In some groups in this classification, more than 40 per cent of respondents wanted to spend less or no time on the work tasks.

When it came to meal preparation, some home care workers wanted to spend more time and others less. However, for all subgroups, at least 20 per cent of home care workers expressed the desire to spend less time with meal preparation. When it came to accompaniment, the results varied. Interestingly, home care workers appeared to be happy to maintain many of the work tasks at their current level. For example, at least 70 per cent of home care workers in all groups were content with the current distribution of help with bathing and dressing.

Although delegated tasks related to drugs are supposed to be performed by assistant nurses, they were performed on a daily basis by 93 per cent (195 of 210) of respondents with a lower level of training. Still, 13 per cent of nursing assistant who do perform them on a daily basis daily would be happy to perform these tasks more often.

### Propensity score diagnostics

The balance of the covariates was improved with the propensity scores. The standardised difference was at most 69% when weightings for the various work tasks were applied to the covariates, as well as age and tenure alone (data not shown). After weighting was applied, the standardised difference was at most 5.7% for the covariates in the model. For tenure and age, in a few instances, the balance remained above the 10% recommended by Austin and Stuart (Austin and Stuart 2015).

## Discussion

Our study suggests that there is a need for increased resources to strengthen home care workers’ capacity for some work tasks and prevent consequent high workloads and negative health consequences. Work tasks performed on a daily basis—such as responding to personal alarms, running errands outside the home, rehabilitation measures and help with bathing—were linked to a higher workload. When comparing employees who performed a given work task on a daily basis with those who did not, lower QALY scores were seen for those who distributed food every day, while daily meal preparation was associated with a higher QALY score. A similar pattern was observed in the pain/discomfort dimension of work tasks. In the anxiety/depression dimension, responding to personal alarms, running errands outside the home and help with bathing were tasks associated with more problems when performed on a daily basis. Therefore, with the exception of rehabilitation measures, work tasks associated with a high workload were also associated with a higher level of anxiety/depression.

Across all groups of home care workers, a desire was expressed for more time to spend on social support, walking and rehabilitation measures. Of these tasks, rehabilitation measures were associated with a higher workload, while social support and walking only had a weak, non-significant association. Home care workers reported that they wanted to spend less time responding to personal alarms, performing domestic chores in the home and running errands outside the home. As previously mentioned, both responding to personal alarms and running errands outside the home were linked to a high workload and anxiety/depression. Our interpretation is that health care workers are likely to experience stress due to the inherent unpredictability of these tasks. In the case of running errands, it is possible that home care workers believe that this should be someone else’s job. This interpretation is backed up by the fact that assistant nurses are more likely to express a desire to spend less time on such tasks. In the case of personal alarms, such unscheduled events may interrupt other activities for an extended period of time while urgent and stressful care measures are taken, such as if a home care recipient has had a fall and/or an ambulance is required. Results concerning the time healthcare workers are prepared to spend on a given work task could be interpreted in two ways; either the home care worker thinks the home care recipient needs more time for this particular intervention, or they feel that their own time is too limited to complete all of the tasks.

It is interesting to note that, while that the level of social support was neither associated with a high workload nor poorer health-related quality of life among home care workers, this is the work task for which most home care workers desired more time. This might be explained by a desire on the part of home care workers to see more resources allocated to reducing loneliness among home care recipients, a problem highlighted in a recent survey conducted by the Swedish National Board of Health and Welfare (the Swedish National Board of Health and Welfare 2022). Daily meal preparation was positively related to health-related quality of life among home care workers, while the opposite was the case for food distribution. An interpretation could be that meal preparation gives personnel a less stressful situation compared with other tasks in the occupation, due to more time with the older person.

In Swedish home care, medical decisions are the responsibility of doctors and registered nurses qualified to prescribe medicines. Nurses may delegate the preparation, administration and delivery of medicines to other personnel (the Swedish National Board of Health and Welfare 2022). It is remarkable that most home care workers report being delegated tasks related to drugs. In fact, in our study, there were no major differences between the percentage of assistant nurses and the percentage of personnel with other healthcare training who frequently performed this task. The only conclusion is that the preparation, administration, and delivery of medicines is often left to personnel without the requisite knowledge and competence concerning drugs and pharmacology. The fact that healthcare interventions are commonly performed by other categories of home care workers than assistant nurses is also confirmed in the official report of a Swedish Government commission of inquiry into strengthening health and social care (Parliament Sweden 2019). In home care services, a lack of competence regarding drugs and pharmacology is clearly a threat to the safety of home care recipients. Assistant nurses are required to remain informed of any amendments to applicable legislation and regulation in their area of expertise.

We have investigated how the current distribution of work tasks differs from the expressed preferences of home care personnel for the future distribution of tasks. For example, assistant nurses, who have the highest level of training among the personnel surveyed, express a desire to spend less time on tasks such as running errands for home care recipients. Swedish municipalities have a statutory right to provide services for residents who are 69 years or older (Parliament Sweden). These services may be provided by the municipality’s own home care organisation or through other providers. Regardless of who provides the service, there is no reason not to reallocate tasks that require no formal training from assistant nurses, leaving them with more time for tasks such as delegated tasks related to drugs.

Our study has some limitations. Almost half of those who received questionnaires did not participate. While, this may have biased results, we feel confident that the results are representative of those with great experience in home care services, as the majority of those who did respond worked at least 30 h a week, were permanently employed (90%) and had worked for at least 5 years in the profession (66%). In our study, we have investigated 15 different work tasks and their relationship with workload and health-related quality of life, thus, resulting in many statistical comparisons. Some of our results, therefore, risk being a result of spurious associations rather than real relationships. Further study can, therefore, be recommended to get a deeper understanding of potential relationships.

A further limitation is that some of the work tasks, such as food distribution, may have been subject to misinterpretation, affecting the validity of our results. However, the survey instrument should at least be able to ensure that home care personnel generally interpret the expressions in a similar manner, mitigating the risk of biased results. To further understand the interpretation of our result, it would be useful to validate how the respondents define the various work tasks.

Propensity scores were used to handle confounding variables. We had a limited number of occurrences of outcomes for age and tenure (these variables were combined in our analyses) with a standardised difference above the level recommended by Austin and Stuart (Austin and Stuart 2015). Considering that we examined 15 work tasks and that we combined these variables to derive one variable, and there were few inconsistencies, the propensity scores served their purpose in removing confounding effects. Our study was limited to northern Sweden, and the results might, therefore, not be generalisable to all other possible settings. However, similar experiences have been found in research conducted in Sweden and other Nordic countries (Trydegård 2012), as well as Switzerland (Möckli et al. 2020). Hence, it is expected that our findings will be highly relevant in other countries with home care systems similar to Sweden’s.

The association between workload and health-related quality of life for home care workers was investigated in a previous study, which showed that a high workload has a negative effect on the health of home care workers (Sjöberg et al. 2020). It is difficult to relate workload and health-related issues with work tasks from other studies. Our study, therefore, contributes unique information and evidence to strengthen the argument for increasing the resources available to home care services. Our study contributes with important information to assess the cost-effectiveness of increased staffing. This has not previously been studied and we recommend that this be done.

Over time, the workload has increased for many occupations (Falkstedt and Hemmingsson 2011; Strandell 2020; Szebehely et al. 2017). Our study gives an insight not only into the distribution of work among home care workers in northern Sweden, but also into the risks associated with allocating insufficient time to perform tasks. The results of which affect both staff personally and the quality of care they can offer home care recipients. It is possible that our results can be explained by personnel being given too little time to perform work tasks, something that appears to cause anxiety and depression. In addition to this, our results provide information about which work tasks can contribute to more pain problems. Another explanation could be that work tasks are assigned on the basis that personnel have limited capacity due to health reasons, e.g. pain problems. This view is supported by home care workers’ preferences for future work allocations. Our study provides a platform for further research on this important topic.

Despite the many challenges faced by home care workers (Grønoset Grasmo et al. 2021a), they also report many positive presence factors in their work, as shown in a meta-synthesis by Grønoset Grasmo et al. (Grønoset Grasmo et al. 2021b). If working conditions improve, skills shortages may not present such a major problem in future (Parliament Sweden 2019), as a more balanced workload among personnel should reduce the number of home care workers leaving the occupation (Van Aerschot et al. 2021).

While a better distribution of work tasks is important for the health and wellbeing of home care personnel, it is also important to consider existing needs-assessment procedures for home care and to what extent proactive approaches could impact the allocation of resources (Zingmark and Bernspång 2011). Older adults who are allocated home care services are often at risk of increasing dependency on that support, For example, a Swedish study shows that, a year after first receiving municipal home care, a large proportion of home care recipients had declined towards increasing levels of dependency (Zingmark and Norström 2021). Thus, improvements in working conditions are also likely to be of high value to the home care recipient. Work tasks related to a high workload in our study can be connected to support that is important to them. Changes to home care services in Sweden have placed a greater burden on the families of home care recipients (Szebehely and Meagher 2018; Ulmanen and Szebehely 2015). Improving the situation for home care personnel could, therefore, have broader societal benefits in addition to increased job satisfaction.

In conclusion, our study contributes useful knowledge for improving working conditions in the over-burdened home care sector. We could connect daily performance on two work tasks, food distribution and meal preparation, to health problems related to QALY and pain/discomfort, but not an increased workload. Responding to personal alarm and domestic chores outside home, suggested an increased workload leading to more problems with anxiety/depression.

The results provide a platform for further exploration of how changes to how tasks are allocated might positively impact workload and health. Offsetting tasks with negative health consequences with tasks that are more positive and meaningful for both the home care worker and home care recipients could lead to improvements within home care. At present, home care services involve a mixture of work tasks that seem to be suboptimal in terms of workload and health consequences. Any development towards an alternative, healthier mix of tasks demands careful consideration of how services are allocated based on current policies and legislation and of how tasks requiring specialist competence can be matched with the appropriate individuals.

## Supplementary Information

Below is the link to the electronic supplementary material.

Supplementary file1 (DOCX 15 KB)

## Data Availability

The datasets generated and/or analysed during the current study are not publicly available because the Swedish Data Protection Act (1998:204) does not permit sensitive data on humans to be freely shared. The datasets are available based on ethical permission from the Regional Ethical board in Umeå, Sweden, from one of the authors (Fredrik Norström).

## References

[CR1] Andersen GR, Westgaard RH (2015). Discrepancies in assessing home care workers’ working conditions in a Norwegian home care service: differing views of stakeholders at three organizational levels. BMC Health Services Research.

[CR2] Andersson K, Kvist E (2015). The neoliberal turn and the marketization of care: the transformation of eldercare in Sweden. Eur J Womens Stud.

[CR3] Austin PC, Stuart EA (2015). Moving towards best practice when using inverse probability of treatment weighting (IPTW) using the propensity score to estimate causal treatment effects in observational studies. Stat Med.

[CR4] Nordic Council of Ministers QPSNordic General Questionnaire for Psychological and Social Factors at Work. In: Nordic Council of Ministers,. https://www.qps-nordic.org/en/doc/QPSNordic_questionnaire.pdf 2022

[CR5] Dallner M, Lindström K, Elo A-L, et al. (2000) Användarmanual för QPSNordic: Frågeformulär om psykologiska och sociala faktorer i arbetslivet utprovat i Danmark, Finland, Norge och Sverige Arbetslivsrapport vol 2000:19, http://www.ammuppsala.se/sites/default/files/fhv-metoder/QPSnordic%20manual.pdf

[CR6] Davison AC, Hinckley DV (1997). Bootstrap Methods and their Application.

[CR7] Devlin NJ, Brooks R (2017). EQ-5D and the EuroQol group: past, present and future. Appl Health Econ Health Policy.

[CR8] Dolan P (1997). Modeling valuations for EuroQol health states. Med Care.

[CR9] Falkstedt D, Hemmingsson T (2011) Hälsokonsekvenser av arbetslöshet, personalneddragningar och arbetsbelastning relaterade till ekonomisk nedgång. Arbetsmiljöverket 2011:11(2015-04-15)

[CR10] Försäkringskassan,  (2018). Sjukfrånvaron på svensk arbetsmarknad - sjukskrivningar längre än 14 dagar och avslut inom 180 dagar i olika branscher och yrken. Socialförsäkringsrapport.

[CR11] Forsberg Kankkunen T, Bejerot E, Björk L, Härenstam A (2014) New Public Management i kommunal praktik. En studie om chefers möjlighet att hantera styrning inom verksamheterna Vatten, Gymnasium och Äldreomsorg. ISM-Rapport, Institutet för stressmedicin 15

[CR12] Grønoset Grasmo S, Liaset IF, Redzovic SE (2021). Home care workers’ experiences of work conditions related to their occupational health: a qualitative study. BMC Health Services Res.

[CR13] Grønoset Grasmo S, Liaset IF, Redzovic SE (2021). Home health aides’ experiences of their occupational health: a qualitative meta-synthesis. Home Health Care Serv Q.

[CR14] Hasson H, Arnetz JE (2008). Nursing staff competence, work strain, stress and satisfaction in elderly care: a comparison of home-based care and nursing homes. J Clin Nurs.

[CR15] Horneij EL, Jensen IB, Holmström EB, Ekdahl C (2004). Sick leave among home-care personnel: a longitudinal study of risk factors. BMC Musculoskelet Disord.

[CR16] Lunceford J, Davidian M (2004). Stratification and weighting via the propensity score in estimation of causal treatment effects: a comparative study. Stat Med.

[CR17] Meagher G, Szebehely M, Mears J (2016). How institutions matter for job characteristics, quality and experiences: a comparison of home care work for older people in Australia and Sweden. Work Employ Soc.

[CR18] Möckli N, Denhaerynck K, De Geest S, Leppla L, Beckmann S, Hediger H, Zúñiga F (2020). The home care work environment’s relationships with work engagement and burnout: A cross-sectional multi-centre study in Switzerland. Health Soc Care Comm.

[CR19] Norström F, Janlert U, Hammarström A (2017). Is unemployment in young adulthood related to self-rated health later in life? Results from the Northern Swedish cohort. BMC Public Health.

[CR20] R Core Team (2015) R: a language and environment for statistical computing. R Foundation for Statistical Computing

[CR21] Sjöberg A, Pettersson-Strömbäck A, Sahlén KG, Lindholm L, Norström F (2020). The burden of high workload on the health-related quality of life among home care workers in Northern Sweden. Int Arch Occup Environ Health.

[CR22] Skovdahl K, Fahlström G, Horttana BM, Winblad B, Kihlgren M (2008). Demanding behaviours and workload in elderly care in Sweden: occurrence at two time points within a decade. Scand J Caring Sci.

[CR23] Smith CJ, Rauhut D (2019). Still “skiing their own race” on new public management implementation? patient choice and policy change in the finnish and swedish health-care systems. Int Rev Adm Sci.

[CR24] Statistics Sweden (2018) Statistical Database, Statistics Sweden. In: Statistics Sweden. https://www.statistikdatabasen.scb.se/pxweb/en/ssd/START__AM__AM0208__AM0208E/YREG50N/ Accessed November 23 2022

[CR25] Strandell R (2020). Care workers under pressure – A comparison of the work situation in Swedish home care 2005 and 2015. Health Soc Care Comm.

[CR26] Swedberg L, Chiriac EH, Törnkvist L, Hylander I (2013). From risky to safer home care: health care assistants striving to overcome a lack of training, supervision, and support. Int J Qual Stud Health Well Being.

[CR27] Sweden P (2019). Stärkt kompetens i vård och omsorg. SOU.

[CR28] Sweden P (2022). Lag Om Vissa Kommunala Befogenheter. SFS.

[CR29] Sweden P (2022). Social services act. SFS.

[CR30] Swedish Association of Local Authorities and Regions (2022) Tabeller Kommunal Personal, Swedish Association of Local Authorities and Regions. In: Swedish Association of Local Authorities and Regions. https://skr.se/download/18.5c6508cf17ff30de8ac15849/1649248305259/Tabell-A-KP-2021.pdf Accessed November 23 2022

[CR31] Szebehely M, Meagher G (2018). Nordic eldercare - Weak universalism becoming weaker?. J Eur Soc Policy.

[CR32] Szebehely M, Trydegård GB (2012). Home care for older people in Sweden: a universal model in transition. Health Soc Care Comm.

[CR33] Szebehely M, Stranz A, Strandell R (2017). Vem ska arbeta i framtidens äldreomsorg?.

[CR34] the Swedish National Board of Health and Welfare (2022) Vad tycker de äldre om äldreomsorgen? 2022-6-7918

[CR35] Trydegård G-B (2012). Care work in changing welfare states: Nordic care workers’ experiences. Eur J Ageing.

[CR36] Ulmanen P, Szebehely M (2015). From the state to the family or to the market? consequences of reduced residential eldercare in Sweden. Int J Soc Welf.

[CR37] Van Aerschot L, Puthenparambil JM, Olakivi A, Kroger T (2021). Psychophysical burden and lack of support: Reasons for care workers' intentions to leave their work in the Nordic countries. Int J Soc Welf.

[CR38] Zingmark M, Bernspång B (2011). Meeting the needs of elderly with bathing disability. Aust Occup Ther J.

[CR39] Zingmark M, Norström F (2021). Transitions between levels of dependency among older people receiving social care - a retrospective longitudinal cohort study in a Swedish municipality. BMC Geriatr.

