# Supplementary material for: How does the distribution of work tasks among home care personnel relate to workload and health-related quality of life?
Source: Int Arch Occup Environ Health. 2023 Jul 12;96(8):1167–81. doi: 10.1007/s00420-023-01997-2 (PMC10504191; doi:10.1007/s00420-023-01997-2)
Supplement: Supplementary file 1 — Supplementary file1 (DOCX 15 KB) [file 420_2023_1997_MOESM1_ESM.docx]

Supplementary file 1.

**Table S1.** Responses to work task and workload questions (n=785-806).

|  |  | **Workload** | |
| --- | --- | --- | --- |
| **Work task** |  | *High* | *Normal* |
| Responding to personal alarm (n=792) | *Daily* | 93 (33%) | 187 (67%) |
|  | *Not daily* | 134 (26%) | 378 (74%) |
| Running errands outside the home (n=802) | *Daily* | 72 (39%) | 113 (61%) |
|  | *Not daily* | 159 (26%) | 458 (74%) |
| Domestic chores in the home (n=804) | *Daily* | 166 (30%) | 393 (70%) |
|  | *Not daily* | 65 (27%) | 180 (73%) |
| Social support (n=795) | *Daily* | 137 (32%) | 296 (68%) |
|  | *Not daily* | 93 (26%) | 269 (74%) |
| Help at toilet visits (n=802) | *Daily* | 196 (29%) | 478 (71%) |
|  | *Not daily* | 36 (28%) | 92 (72%) |
| Dressing (n=803) | *Daily* | 218 (30%) | 499 (70%) |
|  | *Not daily* | 15 (17%) | 71 (83%) |
| Food distribution (n=801) | *Daily* | 180 (29%) | 448 (71%) |
|  | *Not daily* | 51 (29%) | 122 (71%) |
| Meal preparation (n=800) | *Daily* | 113 (29%) | 272 (71%) |
|  | *Not daily* | 117 (28%) | 298 (72%) |
| Feeding, practical help with meals (n=797) | *Daily* | 112 (31%) | 246 (79%) |
|  | *Not daily* | 117 (27%) | 322 (73%) |
| Rehabilitation efforts (n=795) | *Daily* | 115 (36%) | 201 (64%) |
|  | *Not daily* | 115 (24%) | 364 (76%) |
| Delegated tasks related to drugs (n=806) | *Daily* | 214 (28%) | 542 (72%) |
|  | *Not daily* | 18 (36%) | 32 (64%) |
| Supervision (n=797) | *Daily* | 191 (28%) | 480 (72%) |
|  | *Not daily* | 36 (29%) | 90 (71%) |
| Accompaniment (n=785) | *Daily* | 24 (28%) | 61 (72%) |
|  | *Not daily* | 198 (28%) | 502 (72%) |
| Walking (n=785) | *Daily* | 60 (33%) | 121 (67%) |
|  | *Not daily* | 172 (27%) | 454 (73%) |
| Help with bathing (n=803) | *Daily* | 108 (35%) | 200 (65%) |
|  | *Not daily* | 125 (25%) | 370 (75%) |
